# Framing Pro-Anorexia Discourse on YouTube in South Korea: Social Network and Exponential Random Graph Model Analysis of Video Communities

**DOI:** 10.2196/77168

**Published:** 2025-11-04

**Authors:** Daseul Oh, Shin Haeng Lee

**Affiliations:** 1 School of Media and Communication, Chung-Ang University, 305 Building, #918, 84, Heukseok-ro, Dongjak-gu, Seoul, 06974, Republic of Korea, +82 28205174

**Keywords:** pro-anorexia, YouTube, framing, echo chamber, social network analysis, exponential random graph model, influencer, recovery narrative, South Korea, health communication

## Abstract

**Background:**

YouTube, as a participatory platform, allows algorithmic curation and user engagement to shape health information flows. This dynamic amplifies and isolates harmful narratives, producing enclosed “refracted publics.” Pro-anorexia (pro-ana) content exemplifies this, glamorizing extreme thinness as self-control and promoting disordered eating while distancing viewers from evidence-based health discourse. Despite concerns about public health consequences, few studies have examined how channel characteristics and framing strategies drive engagement and echo chamber formation.

**Objective:**

This study analyzed how pro-ana discourse circulates on YouTube and identified entry points for intervention. It (1) compared pro-ana, anti–pro-ana, and recovery frames by channel type (institutional vs individual) and subscriber scale (mega [≥1,000,000], meso [100,000-999,999], or micro [10,000-99,999]); (2) constructed video-level networks from overlapping commenters to trace discourse clustering over time; and (3) evaluated how frame and channel attributes shape network connectivity and frame-crossing.

**Methods:**

We collected 489 Korean-language YouTube videos (January 2020-August 2024) using pro-ana keywords and related-video crawling. Each was coded into 3 frames. Channels were classified by operator type and subscriber scale. A weighted commenter-overlap network was built, backbone-extracted, and analyzed through social network analysis. An exponential random graph model tested predictors of intervideo ties.

**Results:**

Of 489 videos, 369 (75.5%) promoted pro-ana content, mostly in micro- and meso-individual channels (361/369, 97.8%). Recovery frames appeared mainly in micro-individual channels (47/52, 90%). Anti–pro-ana frames clustered in institutional channels (46/68, 68%) but remained structurally peripheral. After backbone filtering, the commenter-overlap network comprised 435 videos and 906 edges. The network showed low density (0.96%) and moderate modularity (0.58) with 19 communities, indicating a fragmented, echo chamber–like structure. Longitudinally, density declined while modularity rose, consistent with echo-chamber intensification. The largest community (n=242) was predominantly pro-ana, while recovery-only (n=25) and anti–pro-ana (n=13) communities were peripheral. The exponential random graph model revealed that homophily was strong for anti–pro-ana (odds ratio [OR]=2.20; *P*<.001) and became significant for pro-ana with channel×frame interactions (OR=3.12; *P*<.001). Critically, recovery frames in meso-individual channels were associated with greater intervideo connectivity (OR=2.59; *P*<.001), and anti–pro-ana frames in those channels were also connective (OR=1.55; *P*=.003).

**Conclusions:**

Pro-ana discourse on YouTube is fragmented into “refracted publics.” Meso-level individual channels using recovery narratives serve as critical bridges across polarized frames. Public health efforts should partner with mid-tier influencers to co-create emotionally resonant recovery content that disrupts echo chambers and fosters cross-frame dialogue.

## Introduction

### YouTube as a Participatory Platform for Health-Compromising Discourse

Digital platforms are now central to public discourse. YouTube strongly influences opinion and social norms through its algorithmic system and creator-driven content [1-3]. Across domains—news, health, education, and politics—it serves as a primary information gateway, bypassing traditional media filters and enabling decentralized participation [4]. Yet, this openness also enables harmful discourse, including misinformation, health-compromising content, and hate speech, threatening public health and social cohesion.

A clear example is “pro-anorexia” (pro-ana) discourse, which glamorizes extreme dieting. These narratives reframe anorexia’s pathological traits as esthetic and moral ideals, creating communities around “thinspiration” images, restrictive meal plans, exercise regimens, and weight-loss stories [5-7]. They strongly affect adolescent females, provoking identity responses. Within these groups, starvation and bodily discipline appear as self-development and social validation tools [8-10]. Although members may gain emotional support and collective identity [5,11-13], they remain vulnerable to anxiety, shame, and self-hatred when failing to meet body norms [14,15].

### Algorithmic Structuring and Fragmentation of Pro-Ana Communities

YouTube recontextualizes pro-ana discourse differently from image-centric platforms like Instagram and Pinterest. Its mixed-genre videos—vlogs, challenges, and reviews—embed “body talk” into everyday narratives [14,16]. In this space, where production and consumption overlap, users develop bonds with creators via comments, likes, and subscriptions. This accelerates discourse cohesion, evolving into “video-based communities” built on shared beliefs [17]. These groups are defined by recurrently consumed, interacted-with videos, forming cognitive and emotional structures beyond playlists or channels—platform-based collectives driven by affect and action.

YouTube’s algorithm strengthens such collectives by analyzing behavioral data—clicks, watch time, comments, and subscriptions—to repeatedly recommend similar content [18,19]. This fosters “filter bubbles” and “echo chambers,” reinforcing preferences while limiting alternative exposure [20,21]. Content with high engagement circulates more widely, amplifying intervideo connectivity through shared audiences [19,22]. Prior research highlights interactivity, repetition, emotional resonance, and network structures as central to shaping these communities [5,23-27].

While YouTube’s algorithmic curation and creator-viewer interactivity foster cohesion among similarly framed content, they hinder bridging across divergent frames [19,28]. Pro-ana advocacy videos often employ emotionally charged personal storytelling, eliciting empathetic engagement that is algorithmically amplified [5,11], which creates closed clusters [22]. In contrast, anti–pro-ana and recovery content, largely from public health perspectives, remains informational and authoritative, making it less effectively engaging and rarely connected to pro-ana communities [2,13,29,30]. Thus, pro-ana content forms dense but isolated clusters.

Within YouTube’s fragmented discursive ecology, pro-ana discourse functions not as a unified sphere but as heterogeneous clusters organized around competing frames. Fragmentation aligns with channel type. Institutional channels, run by news outlets or agencies, deliver authoritative critiques of disordered behaviors using credibility and infrastructure [31-33]. Conversely, many individual creators—neither health professionals nor public figures [34,35]—share autobiographical narratives that glorify thinness or describe recovery, fostering emotional immersion and parasocial bonds, and sometimes integrate monetization strategies [1,2,36,37].

### Strategic Role of Recovery Frames and Meso-Level Channels

Subscriber scale influences video visibility, framing styles, and engagement. “Meso-level” creator channels—moderate in subscribers—often balance reach and intimacy. They outperform large-scale channels in emotional resonance and exceed micro-channels in visibility, making them potential “bridges” for disrupting clusters and introducing alternative frames [38,39]. Exploring how these channels foster frame intersection is crucial for mitigating pro-ana health risks.

Few studies have assessed the structural organization of pro-ana discourse on YouTube or strategies for transforming it into intersectable networks. Prior work has emphasized social support and emotional bonds from pro-ana messaging, showing advocacy’s appeal over prevention [9,29] while noting the marginalization of recovery narratives [30]. Yet, most analyses have focused on individual video content, neglecting intervideo ties and frame diffusion from a network perspective [40,41].

Accordingly, this study analyzed network-level relationships among pro-ana advocacy, anti–pro-ana, and recovery frames on YouTube. It investigated whether pro-ana discourse is structurally enclosed within platform clusters and explored strategies for more effective public health messaging. Specifically, we assessed whether recovery or anti–pro-ana narratives generate wider engagement and how they operate within the broader video network.

We emphasized the strategic value of the “recovery frame,” often conveyed through autobiographical confessions by former pro-ana individuals. Such narratives reframe eating disorders as emotionally resonant journeys, blending information and affective appeal [40,42]. With this hybrid structure, recovery frames can align with algorithmic preference while retaining emotional pull [14,41]. We tested whether recovery narratives, delivered through suitable channels, soften boundaries and enable cross-frame interaction.

This study situated the analysis in Korea, where idol-driven thinness ideals and self-discipline ethics strongly shape young women’s body norms [43]. It diagnosed structural isolation among pro-ana frames and evaluated recovery’s potential for diffusion. This will expand theories of platform-based health risk communication and guide youth protection strategies, including algorithm adjustment, targeted monitoring, and channel-specific messaging. Ultimately, the study will advance communication models that address emotionally driven, closed discourse communities.

### Research Questions

In the digital era, health discourse is no longer expert-led and unidirectional. On participatory platforms like YouTube, algorithmic curation and user activity drive sophisticated, fragmented modes of dissemination and transformation. Within this environment, pro-ana discourse—glamorizing pathological body images—emerges as a public health threat. Understanding how it spreads and evolves structurally is urgent. This study examined how pro-ana discourse is produced and consumed through framing strategies and how these frames form distinct network structures shaped by algorithms and engagement.

As the first research question (RQ1), this study analyzed framing differences by channel operator (institutional vs individual) and subscriber scale (mega, meso, or micro). Unlike prior studies that focused on video content or anecdotal cases [5,11,13,40], this study empirically assessed how channel structure and scale influence frame selection and organization. This approach highlights discourse diversity and differentiation while offering insights for targeted public health interventions.

The second research question (RQ2) explored how pro-ana–related videos form structural networks through viewer engagement and how these networks evolve. It examined how channel attributes and framing strategies align within viewer-driven video networks. A video-level network was built using commenter overlap to define intervideo ties. Structural indicators—density, modularity, and community count—were analyzed longitudinally. This study tested the claim that pro-ana discourse comprises multiple “refracted publics” rather than a unified sphere [1]. It also assessed whether algorithms and user activity create increasingly enclosed, fragmented structures. As homogeneity strengthens, networks are expected to cluster tightly, with dense internal ties and sparse external links—hallmarks of echo chambers [44].

The third research question (RQ3) examined how channel characteristics and frame attributes influence intervideo connections using the exponential random graph model (ERGM). Beyond descriptive engagement, the ERGM evaluates when frame-based homophily strengthens and when heterogeneous ties capable of bridging emerge. Special focus was given to meso-level individual channels, which—with moderate subscriber bases—may act as bridges when paired with anti–pro-ana or recovery frames. By identifying these roles, this study aimed to develop strategies for frame-channel collaboration in public health communication to counter the structural fragmentation and closure of pro-ana discourse on YouTube.

## Methods

### Ethical Considerations

This study analyzed only publicly available YouTube videos and comments; no private content was included. Public user handles were used only when necessary; however, all handles were deidentified in any shared datasets and were not publicly released. Data collection complied with the YouTube Data API Terms of Service and relevant legal guidelines. Researchers had no direct interaction with users. Since the study used noninterventional, publicly accessible, nonidentifiable data with minimal risk, no formal ethics review was required.

### Data Collection

To analyze Korean-language YouTube discourse on eating disorders and extreme dieting, we used a multistage strategy in August 2024 via the YouTube Data API (v3). The procedure included (1) exploratory seed video retrieval, (2) expansion through algorithmically recommended videos, and (3) topic-based filtering and refinement.

#### Stage 1: Seed Video Retrieval

We identified seed videos using 4 Korean search terms linked to pro-ana and extreme dieting: “프로아나” (pro-ana), “프아 다이어트” (pro-ana diet), “뼈말라” (ppyeo-malla; “bone-thin”), and “개말라” (gae-malla; “extremely skinny”). These keywords were drawn from prior studies [13,41] and discourse analyses in Korean online communities. Using the YouTube Data API’s *search.list* endpoint, we retrieved top-ranked videos for each keyword by relevance. Videos explicitly tagged or described as pro-ana or extreme dieting formed the initial seed sample.

#### Stage 2: Expansion via Related Videos

To capture YouTube’s algorithmic exposure, we collected “related videos” for each seed video from the sidebar and autoplay suggestions. Because recommendations depend on viewing history, we used a clean, no-history account—free of logins, subscriptions, or prior views—to scrape related data. This reduced personalization bias and more objectively reflected default exposure chains [22].

#### Stage 3: Sample Refinement

The initial corpus included 4174 videos. We refined the sample by (1) restricting publication to the period from January 2020 to August 2024, aligning with pandemic-linked increases in youth disordered eating [45]; (2) removing duplicates and videos without comment sections; and (3) excluding off-topic content after reviewing titles, descriptions, and transcripts. Videos without pro-ana advocacy, anti–pro-ana critique, or recovery promotion were discarded. The final dataset comprised 489 videos from 160 channels. Metadata included video ID, view count, comment count, channel name, and subscriber count (Multimedia Appendix 1).

### YouTube Channel Classification

To evaluate how channel traits influence frame choice and network structure, we classified channels along 2 dimensions: operational entity (individual vs institutional) and subscriber size (mega, meso, or micro). This scheme reflects institutional embeddedness and communicative reach, providing meaningful strata for analyzing framing strategies and network positions.

#### Operational Entity

Channels were coded as *institutional* or *individual*. Institutional channels included those managed by news outlets, broadcasters, health organizations, hospitals, public agencies, celebrity agencies, or corporations. These typically display organizational names and standardized formats. Individual channels, operated by single creators, often feature vlogs, diet routines, or testimonials, and may share personal contact details for sponsorship. This distinction captures ownership, production intent, trustworthiness, audience expectations, and algorithmic visibility [1].

#### Subscriber Size

Subscriber count, a proxy for social influence and algorithmic visibility, was divided into 3 tiers based on influencer marketing literature [1,37]: *mega* (≥1,000,000 subscribers), *meso* (100,000-999,999 subscribers), and *micro* (10,000-99,999 subscribers).

Combining the 2 dimensions produced 6 channel types: mega-institution, mega-individual, meso-institution, meso-individual, micro-institution, and micro-individual. Meso-individual channels held the largest share (302/489, 61.8%), followed by micro-individual channels (129/489, 26.4%), showing that small- to mid-sized individual creators drive pro-ana discourse on Korean YouTube (Table 1).

**Table 1.** Channel and video characteristics by channel category in YouTube pro-anorexia discourse (2020‐2024).

Category	Channels (N=160), n (%)	Average subscribers, n	Videos (N=489), n (%)	Average views, n
Mega-institution	21 (13.1)	2,676,190	26 (5.3)	1,216,276
Mega-individual	3 (1.9)	2,220,000	4 (0.8)	2,980,878
Meso-institution	19 (11.9)	487,211	20 (4.1)	645,684
Meso-individual	35 (21.9)	335,057	302 (61.8)	706,627
Micro-institution	7 (4.4)	43,864	8 (1.6)	214,262
Micro-individual	75 (46.9)	14,472	129 (26.4)	246,226

Mega-individual channels, though fewer, recorded the highest average views per video, indicating disproportionately broad reach (Table 1). In contrast, meso- and micro-individual channels were central to grassroots production and engagement, making them structurally pivotal for network propagation. These classifications were later used as independent variables to explain frame distribution (RQ1), community structure (RQ2), and edge formation (RQ3).

### Frame Analysis for RQ1

To address RQ1, we analyzed 489 YouTube videos to examine tensions between pro-ana narratives and public health counter-responses. Frames are defined here as interpretive structures that shape audiences’ problem recognition, emotions, and attitudes by guiding how issues are presented [46,47]. While traditional media once centralized frame dissemination, digital media now enables YouTubers to influence audiences through algorithmically optimized strategies [22,48]. Creators often use sensational or boundary-setting discourse to define “normal” bodies and acceptable behavior [49]. In health-risk discourses like pro-ana, frames serve not just as information vehicles but also as mechanisms for belief formation and identity performance [34,35]. When algorithmically reinforced, frame fragmentation intensifies polarization [1,18].

Based on prior studies, we categorized 3 major frames in pro-ana YouTube content. The *pro-ana advocacy frame* glamorizes extreme weight loss and presents thinness as ideal beauty, portraying practices as self-discipline or growth. Examples include diet vlogs, body-check challenges, and calorie tutorials, often embedded in the narratives of effort and transformation [41]. Creators position themselves as role models through microcelebrity strategies, embodying beauty and willpower. Their emotional bonds with followers reproduce the pro-ana frame [7,10].

In contrast, the *anti–pro-ana frame* stresses the dangers of extreme dieting and the harms of pro-ana narratives. It appears mainly in institutional channels, such as news media or expert-led accounts, using formats like news clips, interviews, and warning narratives. These videos critique sociocultural pressures that valorize thinness, reject normalization of pro-ana discourse, and emphasize the ethical need for intervention [40,41].

Lastly, the *recovery frame* centers on personal recovery from eating disorders, such as food diaries and mental health stories. These autobiographical accounts evoke empathy and identification among viewers, introducing decentered voices that disrupt pro-ana communities [40,42]. Expressive strategies include autobiographical narration, contrastive references to past videos, and audience feedback loops.

The frame typology was operationalized through a coding scheme as presented in Table 2, which outlines subframes under each primary frame. Subframes were coded using a multi-label strategy and aggregated under the primary frame. When multiple frames appeared in a video, dominance was determined by subframe frequency, with coder agreement resolving inconsistencies.

**Table 2.** Operational definitions and examples of pro-ana^a^ video frames.

Main frame and subframe		Definition	Example	Prior studies
Pro-ana advocacy				
Thinness glorification		Frames extreme thinness as beauty and an aspirational value.	Idolizing emaciated celebrity bodies and sharing strict diet before-after images.	[11,40]
Self-control		Presents diet and exercise as essential practices for achieving an ideal body.	Framing fasting or intense workouts as a healthy discipline.	[11,40]
Self-harm		Links thinness pursuit to fear, guilt, and harmful behaviors.	Linking weight-loss failure to anxiety and shame.	[11,40]
Anti–pro-ana				
Disruption of everyday life		Positions anorexia as a disorder threatening health and daily life.	Depicts isolation, family strain, or health crises caused by disordered eating.	[41]
Personal responsibility		Attributes pro-ana practices to the personal pursuit of thinness.	Criticizes young people who imitate celebrity thinness for self-gratification.	[41]
Societal responsibility		Blames cultural and media pressures (eg, idol culture and social media) for promoting pro-ana norms.	Argues that idol-driven beauty norms fuel eating disorders among young women.	[41]
Recovery				
Restoration of everyday life		Highlights return to normal eating and daily routines after recovery.	Sharing meal plans or journals documenting healthy weight recovery.	[40,42]
Self-reflection		Promotes body acceptance, regret over harmful behaviors, and hope for future well-being.	Recalling past self-harm while expressing renewed motives for self-care.	[40,42]

a pro-ana: pro-anorexia.

The coding team included 2 master’s students in media studies. After a pilot phase refining definitions and examples, independent coding was applied to all 489 videos. Intercoder reliability was assessed by double-coding 154 random videos (31% of the total), yielding Cohen κ values of 0.78-0.83 across frames, which indicate substantial agreement and coding validity.

### Social Network Analysis for RQ2

To address RQ2, we used social network analysis (SNA) to examine how pro-ana videos are linked through shared commenters and how these links reflect temporal shifts in discursive cohesion and fragmentation. A video-level network was built from commenter overlap. Using the *commentThreads* endpoint of the YouTube Data API, we collected comments and user IDs from the 489 videos. The dataset included 1,21,991 comments (range 1‐4935; mean 254.5, SD 411.2), providing a large sample for interaction analysis (Multimedia Appendix 2).

#### Stage 1: Network Construction

We built a 2-mode (video×user) affiliation matrix linking each video to its top commenters and then projected it onto a 1-mode (video×video) network. An edge between videos A and B was created if at least one user commented on both [19]. To reduce distortions from popular videos or high-subscriber channels [50], edge weights were defined as the proportion of commenters on video A who also commented on video B, normalizing for exposure scale [19].

#### Stage 2: Backbone Extraction

To retain only statistically meaningful ties in the skewed comment network, we applied the disparity filter, which preserves edges exceeding random expectation thresholds (*P*<.05) [51]. This extracted the network’s significant backbone, isolating structural ties indicative of discourse cohesion.

#### Stage 3: Community Detection and Structural Indicators

On the backbone network, we calculated global metrics, such as density, community count, and modularity, to assess cohesion. The Louvain algorithm was used for community detection [26]. Modularity values show how well the network is divided into modules with dense internal and sparse external ties [27]; higher values indicate echo chamber–like clusters resistant to frame crossover.

#### Stage 4: Time-Series Network Dynamics

To capture structural change over time, we split the dataset into quarterly periods and repeated backbone extraction, metric calculation, and community detection for each. This longitudinal approach tracked network shifts before and after the COVID-19 pandemic.

### ERGM Analysis

To address RQ3, we used the ERGM to identify factors explaining why pro-ana YouTube videos are connected via shared commenters. Unlike descriptive SNA, which characterizes observed structures, the ERGM models the likelihood of edge formation from node attributes and structural dependencies [25]. This method is effective for revealing structural conditions driving public health risks, focusing not on individual content but on how information is linked, isolated, or diffused [51].

The dependent variable was a binary indicator: 1 when two videos shared at least one commenter, and 0 otherwise, based on the backbone-pruned video network. This captured the presence of a narrative contagion path from a user-driven perspective.

Explanatory variables included channel type and dominant frame. Channel types were grouped into 6 categories based on ownership (institutional vs individual) and subscriber scale (mega, meso, or micro). Frames were coded as pro-ana, anti–pro-ana, or recovery. All variables were categorical.

To capture echo chamber dynamics, we added a *nodematch* term for frame homogeneity (whether connected videos shared the same frame). We also modeled interaction terms between channel type and frame to test whether specific combinations were more likely to produce user overlap and cross-frame ties.

Controls included (1) absolute difference in video views (log-transformed), (2) same-channel indicator (dummy variable), and (3) upload date gap in days. These accounted for exposure scale, channel ownership effects, and temporal proximity. Auxiliary models also included the *GWESP* (Geometrically Weighted Edgewise Shared Partners) term to capture transitivity, ensuring robustness of main effects.

The model was estimated using the ergm package in R via Markov chain Monte Carlo maximum likelihood estimation [52]. Convergence was checked with trace plots and autocorrelation functions, adjusting tuning parameters, such as burn-in, thinning, and step size, to stabilize standard errors. Coefficients were reported in log-odds and converted into odds ratios for interpretability.

Model fit was assessed through simulation-based goodness-of-fit tests comparing observed and simulated statistics (degree distribution, geodesic distances, and shared partners). Robustness was tested by (1) varying the backbone filter’s α level (*α*=.01, .05, or .10), (2) replicating the model on a binarized unfiltered weighted network, and (3) applying identical model specifications across quarterly networks to compare pre- and postpandemic structural change.

**Figure 1.**
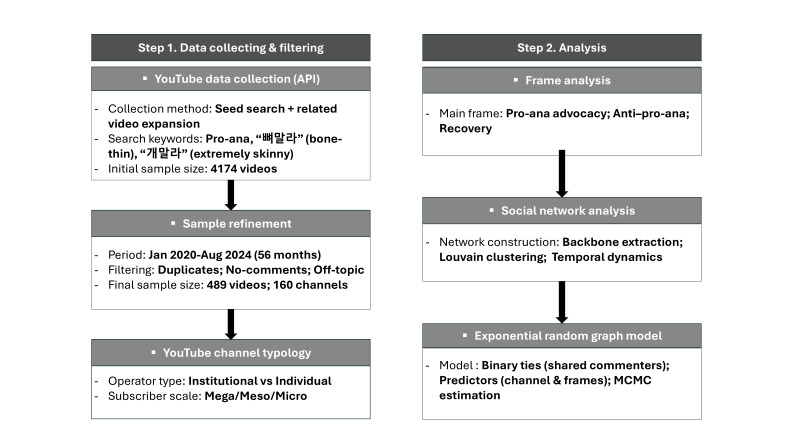
Research flow. API: application programming interface; MCMC: Markov chain Monte Carlo; pro-ana: pro-anorexia.

Our analytical framework (Figure 1) moves beyond video-level content evaluation by statistically identifying combinations of content and structural conditions that facilitate discourse diffusion. It also highlights strategic public health messaging targets within emotionally driven, algorithmically reinforced discourse environments.

## Results

### Use of Frames by Channel Type

We cross-tabulated 8 subframes of pro-ana discourse across 6 channel types (Table 3) and analyzed their distributions. Because videos may include multiple subframes—potentially violating independence assumptions of the chi-square test—and some cells had low counts, we used Monte Carlo–approximated Fisher exact tests by frame. As a sensitivity check, a Monte Carlo chi-square test was also applied to the full table, confirming a highly significant association between subframes and channel type (Monte Carlo *χ*^2^_35_=585.12; *P*<.001).

**Table 3.** Distribution of pro-ana^a^ subframes in 489 YouTube videos by channel type (2020-2024).

Main frame and subframe	Mega-level		Meso-level		Micro-level	
	Institution^b^ (N=26), n (%)	Individual^b^ (N=4), n (%)	Institution^b^ (N=20), n (%)	Individual^b^ (N=302), n (%)	Institution^b^ (N=8), n (%)	Individual^b^ (N=129), n (%)
Pro-ana advocacy						
Thinness glorification	2 (7.7)	1 (25.0)	2 (10.0)	61 (20.2)	0 (0.0)	51 (39.5)
Self-control	2 (7.7)	3 (75.0)	2 (10.0)	276 (91.5)	0 (0.0)	44 (34.1)
Self-harm	1 (3.9)	0 (0.0)	0 (0.0)	6 (2.0)	0 (0.0)	5 (4.0)
Anti–pro-ana						
Disruption of everyday life	20 (76.9)	0 (0.0)	16 (80.0)	8 (2.7)	6 (75.0)	10 (7.8)
Personal responsibility	7 (26.9)	1 (25.0)	6 (30.0)	0 (0.0)	6 (75.0)	7 (5.4)
Societal responsibility	13 (50.0)	0 (0.0)	9 (45.0)	1 (0.3)	4 (50.0)	3 (2.3)
Recovery						
Restoration of everyday life	2 (7.7)	0 (0.0)	1 (5.0)	2 (0.7)	0 (0.0)	44 (34.1)
Self-reflection	2 (7.7)	0 (0.0)	1 (5.0)	2 (0.7)	0 (0.0)	12 (9.3)

a pro-ana: pro-anorexia.

b As multiple subframes could be assigned to a video, totals for each channel type may exceed the number of videos in that category.

Pro-ana advocacy subframes (*thinness glorification*, *self-control*, and *self-harm*) were concentrated in individual channels, especially meso- and micro-individual channels. Standardized residuals showed that *self-control* was far more frequent in meso-individual channels (z=14.95), while *thinness glorification* was more frequent in micro-individual channels (z=4.30). These results suggest that mid- and small-scale channels incubate messages glorifying thinness and reframing disordered eating as “self-discipline.”

Anti–pro-ana subframes (*disruption of everyday life*, *personal responsibility*, and *societal responsibility*) were concentrated in institutional channels. Mega- and meso-institutional channels exceeded expectations for *disruption of everyday life* (z=7.85 and 7.27, respectively), and both *personal responsibility* and *societal responsibility* were overrepresented in mega-institutional channels (z=3.64 and 7.52, respectively) and meso-institutional channels (z=3.74 and 5.82, respectively). These findings confirm that legacy media and professional channels drive critical discourse on eating disorder risks and societal responsibilities.

Recovery subframes, especially *restoration of everyday life* and *self-reflection*, appeared mainly in micro-individual channels. *Restoration of everyday life* was significantly above expectation (z=10.15; *P*<.001), with *self-reflection* also elevated (z=4.03; *P*<.001). This suggests that intimate, recovery-oriented narratives are most clearly articulated by a small-scale creator.

When subframes were aggregated into 3 primary frame categories and reanalyzed (Table 4), channel type and frame category again showed a significant association (*χ*^2^_10_=397.10; *P*<.001). Post hoc residuals indicated strong overrepresentation of pro-ana advocacy in meso-individual channels (z=13.86); anti–pro-ana in mega- (z=10.71) and meso-institutional channels (z=8.72); and recovery in micro-individual channels (z=11.08).

**Table 4.** Frequency of videos (N=489) featuring pro-ana^a^ frames by YouTube channel type.

Variable	Mega-level		Meso-level		Micro-level	
	Institution (N=26), n (%)	Individual (N=4), n (%)	Institution (N=20), n (%)	Individual (N=302), n (%)	Institution (N=8), n (%)	Individual (N=129), n (%)
Pro-ana advocacy	2 (7.7)	3 (75.0)	3 (15.0)	292 (96.7)	0 (0.0)	69 (53.5)
Anti–pro-ana	22 (84.6)	1 (25.0)	16 (80.0)	8 (2.6)	8 (100.0)	13 (10.1)
Recovery	2 (7.7)	0 (0.0)	1 (5.0)	2 (0.7)	0 (0.0)	47 (36.4)

a pro-ana: pro-anorexia.

These results reveal a bifurcated ecosystem: mid-scale individuals diffuse pro-ana advocacy, large institutional channels anchor anti–pro-ana critique, and small individual creators host recovery narratives. Recovery’s concentration in micro-individual channels suggests limited diffusion, underscoring the need for bridging strategies to broaden reach.

### Community Structure of the Pro-Ana Video Network

After backbone filtering, we built a commenter-overlap network with 435 videos and 906 edges representing shared audiences (Table 5). Node degrees ranged from 1 to 247, with a median of 1, showing that most videos shared audiences with only a few others. Network density was low (0.96%). Louvain community detection identified 19 modules, with modularity at 0.58, suggesting closed subgroups with strong internal ties but sparse external links. Thus, pro-ana discourse resembles a fragmented ecology of isolated modules rather than a unified sphere.

**Figure 2.**
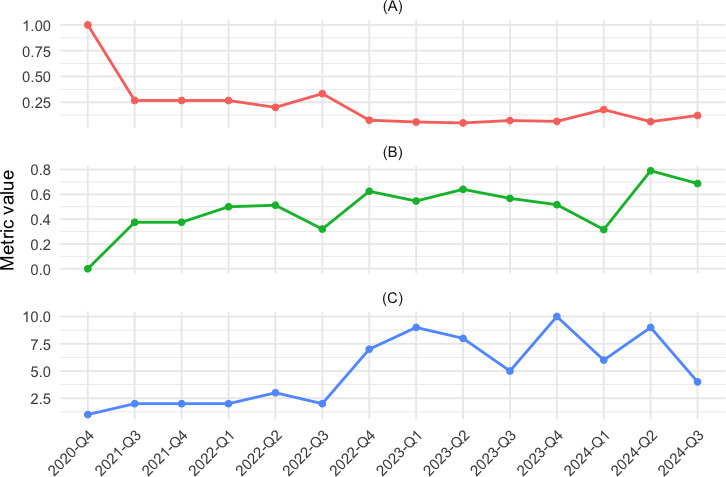
Quarterly trends in structural properties of the pro-anorexia (pro-ana) YouTube commenter network (2020‐2024). This figure illustrates quarterly trends in density (A), modularity (B), and community (C) count for the pro-ana YouTube commenter-overlap network from Q4 2020 to Q3 2024. The network is constructed by linking videos based on shared commenters, with backbone extraction applied to retain only statistically significant connections. Network density reflects the concentration of comments, modularity measures the strength of clustering, and the number of communities indicates the degree of network fragmentation. Quarters with no surviving edges after backbone extraction are excluded.

To extend this static snapshot, quarterly networks were analyzed (Figure 2) for density, modularity, and community count. Density was higher in late 2020 but fell sharply after mid-2021 (Kwiatkowski-Phillips-Schmidt-Shin [KPSS] *P*=.049), showing weakened audience overlap. Modularity increased across the period (KPSS *P*=.046), indicating echo chamber intensification as similar-frame videos increasingly shared commenters. Community counts peaked in Q4 2022 but fluctuated without a clear trend (KPSS *P*=.07). Overall, the postpandemic network grew more polarized into homogeneous, self-reinforcing clusters.

**Table 5.** Backbone commenter-overlap network derived from 489 pro-ana^a^ YouTube videos.

Term	Definition	Value in the observed network
Node	Fundamental network unit; here, each node represents a single pro-ana YouTube video.	435
Edge	Connection between 2 nodes, indicating meaningful commenter overlap—ie, at least a minimum number of shared commenters.	906
Degree	Number of edges incident on a node, showing how many videos are directly linked through shared commenter activity.	Range: 1-247; median=1
Density	Proportion of observed to possible edges, measuring overall connectivity.	0.96%
Modularity (number of communities)	Degree to which the network decomposes into dense internal ties and sparse external ties; includes the number of detected communities.	0.58 (19)

a pro-ana: pro-anorexia.

**Figure 3.**
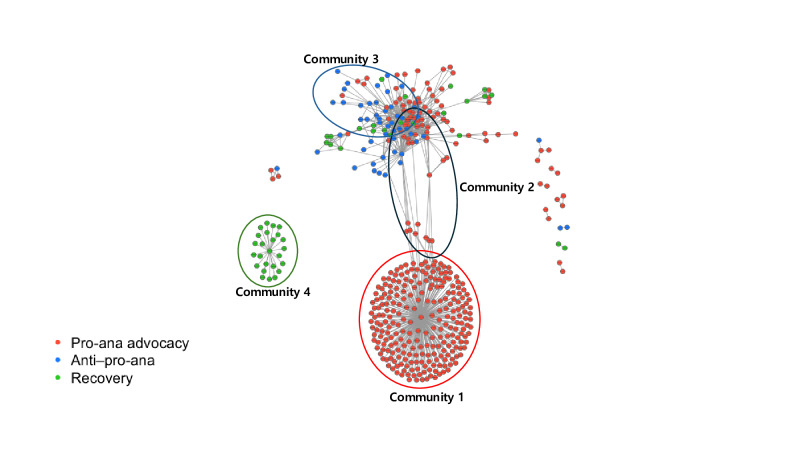
Frame-based community structure of the pro-anorexia (pro-ana) video network on YouTube (2020‐2024). This network visualization illustrates the community structure of the pro-ana video network, based on commenter overlap among 489 YouTube videos from 2020 to 2024. Each node represents a video, and edges connect videos that share common commenters. Colors indicate the dominant frame type of each video: red for pro-ana advocacy, blue for anti–pro-ana, and green for recovery. The visualization highlights frame-based clustering and potential echo chambers within the pro-ana discourse on YouTube.

To analyze thematic content, we examined frame composition in the largest communities (Figure 3). Community 1 (n=242) was almost entirely pro-ana, forming a dense core repeatedly drawing active commenters. Community 2 (n=79) mixed pro-ana, anti–pro-ana, and recovery frames, creating a more heterogeneous discursive space. By contrast, community 3 (n=13) was nearly all anti–pro-ana, and community 4 (n=25) was entirely recovery—both located at the periphery. These results suggest that critical and recovery messages occasionally penetrate the advocacy core but remain marginal, largely confined to peripheral enclaves. Overall, the pro-ana ecosystem is multitiered, dominated by an advocacy-centered core, with critical or recovery frames limited to sporadic bridging.

### ERGM Estimates

For RQ3, we estimated 2 ERGMs with commenter-overlap edges as the dependent variable (Table 6). Model 1 included main effects for channel type, video frame, and frame-based homophily, and controls for log-transformed view count difference and same-channel membership. Model 2 added interaction terms between channel type and frame. Both models converged successfully and fit significantly better than a baseline edges-only model (model 1 Δdeviance=1109.04; *P*<.001).

**Table 6.** Exponential random graph models predicting edge formation in the anorexia-related YouTube video network by channel and frame type.

Variable	Model 1^a^	Model 2^a^
Edges	−4.88^b^ (0.26)	−5.41^b^ (0.33)
Individual terms		
Channel type (reference: micro-personal)		
Mega-institution	1.41^b^ (0.12)	1.80^b^ (0.22)
Mega-individual	1.78^b^ (0.18)	2.11^b^ (0.21)
Meso-institution	1.12^b^ (0.12)	1.31^b^ (0.23)
Meso-individual	−0.75^b^ (0.09)	−1.10^b^ (0.13)
Micro-institution	1.32^b^ (0.17)	1.82^b^ (0.53)
Video frame (reference: pro-ana^c^ advocacy)		
Anti–pro-ana	−0.12 (0.23)	0.40 (0.29)
Recovery	0.29 (0.18)	0.37 (0.29)
Dyadic terms		
Homophily		
Anti–pro-ana	0.79^b^ (0.24)	0.92^d^ (0.33)
Recovery frame	0.55 (0.28)	0.56 (0.40)
Pro-ana advocacy	0.40 (0.23)	1.14^b^ (0.31)
Controls		
View count (absdiff)	−0.77^b^ (0.07)	−0.78^b^ (0.07)
Channel ID (nodematch)	1.72^b^ (0.13)	2.19^b^ (0.16)
Interaction terms		
Mega-institution×anti–pro-ana frame	—^e^	−0.40^f^ (0.16)
Mega-individual×anti–pro-ana frame	—	−0.64^f^ (0.26)
Meso-institution×anti–pro-ana frame	—	−0.24 (0.17)
Meso-individual×anti–pro-ana frame	—	0.44^d^ (0.15)
Micro-institution×anti–pro-ana frame	—	−0.44 (0.33)
Mega-institution×recovery frame	—	−0.03 (0.22)
Mega-individual×recovery frame	—	−0.65 (0.76)
Meso-institution×recovery frame	—	−0.22 (0.29)
Meso-individual×recovery frame	—	0.95^b^ (0.23)
Micro-institution×recovery frame	—	−0.06 (0.49)
Model AIC^g^	9566	9545
Reduction in residual deviance (*df*)		
Compared with the edge-only model	1109.04 (12)^b^	—
Compared with model 1	—	40.99 (10)^b^

a Results are from 2 exponential random graph models testing factors influencing commenter-overlap edges among anorexia-related YouTube videos.

b *P*<.001.

c pro-ana: pro-anorexia.

d *P*<.01.

e Not applicable.

f *P*<.05.

g AIC: Akaike information criterion.

In model 1, relative to micro-individual channels, videos from mega-institutional (β=1.41; *P*<.001; odds ratio [OR]=4.1), mega-individual (β=1.78; *P*<.001; OR=5.9), meso-institutional (β=1.12; *P*<.001; OR=3.1), and micro-institutional channels (β=1.32; *P*<.001; OR=3.8) showed a significantly higher likelihood of commenter sharing. By contrast, meso-individual channels had a significant negative effect (β=−0.75; *P*<.001; OR=0.47). While frame main effects were nonsignificant, strong homophily emerged for anti–pro-ana (β=0.79; *P*<.001; OR=2.20), meaning same-frame videos were more likely to share commenters. Pro-ana and recovery homophily were positive but nonsignificant. Among controls, larger view-count gaps reduced edge probability (β=−0.77; *P*<.001; OR=0.46), while same-channel videos were much more connected (β=1.72; *P*<.001; OR=5.58).

Model 2, with interaction terms, improved fit (Δdeviance=40.99; *P*<.001). Pro-ana homophily became significant (β=1.14; *P*<.001; OR=3.12). The meso-individual×anti–pro-ana interaction was positive (β=0.44; *P*=.003; OR=1.55), showing that mid-scale creators of anti–pro-ana content had greater commenter overlap than expected. The meso-individual×recovery interaction was even stronger (β=0.95; *P*<.001; OR=2.59), indicating that recovery narratives from meso-level creators act as effective bridges across segmented networks. In contrast, the mega-institution×anti–pro-ana (β=0.40; *P*=.01; OR=0.67) and mega-individual×anti–pro-ana interactions (β=−0.64; *P*=.01; OR=0.53) were negative, suggesting that critical content from large channels generated less commenter sharing than predicted.

In summary, channel type, frame, and their interaction significantly shaped commenter-based intervideo connections. Mega-institutional channels showed high audience overlap, forming dense network regions, whereas meso-individual channels with anti–pro-ana and recovery frames acted as connectors, suggesting alternative diffusion paths. These findings indicate that mid-scale creators occupy strategic positions for exposing advocacy-dominated communities to critical and recovery narratives.

## Discussion

### Principal Findings

This study examined pro-ana discourse on YouTube within South Korea’s public health context, where adolescent dieting is a major concern. We analyzed how discourse is structured by channel type and framing strategy, and how overlapping commenters influence network cohesion or fragmentation.

Channels were grouped by ownership and subscriber scale into 6 types, while videos were coded into 3 frames. Analytically, we described framing variation across channel types (RQ1), used SNA to map clustering based on shared commenters (RQ2), and applied the ERGM to test edge formation by frame and channel (RQ3).

The content analysis showed that pro-ana advocacy frames were concentrated in individual-run channels, particularly meso- and micro-level creators. In contrast, institutional channels—especially those with mega or meso followings—primarily used anti–pro-ana frames stressing health risks and social responsibility. Recovery framing appeared most often in micro-level individual channels, underscoring the need to examine how such narratives spread and connect within the broader video network.

Network analysis revealed that the video network consisted of multiple tightly knit clusters. Over time, density declined while modularity increased, indicating growing fragmentation. The largest cluster was dominated by pro-ana advocacy videos, whereas anti–pro-ana and recovery content remained peripheral. Cross-frame bridges existed but were limited in both frequency and strength.

ERGM results showed that institutional channels with mega- or meso-scale audiences were more likely to be linked through shared commenters. Meso-level individual channels, though less connected overall, had significantly higher tie formation when paired with anti–pro-ana or recovery frames. This indicates the potential for bridging discourse clusters, though not causal transmission. Overall, channel type, framing, and their interaction significantly shaped the probability of intervideo connections, highlighting mid-scale individual channels as strategic for spreading health-oriented counter-narratives.

Together, the 3 analyses provide a multidimensional account of how pro-ana discourse is organized and sustained on YouTube. Videos using critical or recovery frames, when disseminated through meso-level individual channels, emerged as key cross-frame junctures. These channels represent strategic nodes for advancing public health communication.

### Implications

This study shows that pro-ana discourse on YouTube does not exist within a single open sphere but rather within a fragmented, multilayered ecology shaped by algorithmic curation and selective viewing—a “refracted public” [1]. Meso- and micro-level individual channels use microcelebrity tactics [53] and platform affordances to promote and normalize extreme dieting. These creators generate concentrated volumes of pro-ana content and foster repeated consumption within niche groups, reinforcing discursive insularity. Conversely, institutional channels emphasize recovery narratives and health warnings, but user preferences and recommendation algorithms restrict their diffusion, limiting them to pre-existing audiences.

Our network analysis using overlapping commenters quantified this segmentation. Rising modularity and declining intercluster connectivity indicate structural limits in linking recovery or critical content with dominant pro-ana clusters. Still, meso-level individual channels emerged as prolific recovery storytellers and bridges for cross-frame commenter overlaps. Their position underscores mid-scale influencers as strategic leverage points for amplifying recovery frame dissemination.

This study advances health communication research by shifting the focus from institutional campaigns or message content alone to the interplay among user participation, network structure, and channel typology. Prior studies have shown that social media reduces stigma and spreads eating disorder information [54,55], and influencers can drive positive change [44,56,57]. Our work adds empirical, network-level analysis of how users engage with and circulate pro-ana frames, offering both theoretical and methodological contributions to platform-based health risk communication.

The Korean context—where thinness ideals are reinforced through fandom, beauty, and fashion content—amplifies the algorithmic visibility of pro-ana discourse. This phenomenon is not unique; similar patterns of esthetic normalization and clustering appear in Western contexts [14,41], suggesting broader applicability of our findings.

Practically, this study offers 3 recommendations for public health authorities and digital health communicators. First, rather than relying on blocking harmful content or top-down criticism, emotionally resonant recovery narratives are needed that align with engagement logics inside pro-ana echo chambers. Collaborating with mid-scale creators—especially those sharing recovery experiences—can reduce resistance and increase receptivity [40,58]. Second, interactive formats that align with YouTube’s affective interface, such as Q&A live streams, recovery-themed challenges, and first-person storytelling, can boost engagement and algorithmic visibility. Third, these strategies extend beyond pro-ana discourse to high-risk contexts like mental health and online extremism, helping transform echo chambers into more interconnected deliberative spaces.

### Limitations

This study focused only on videos with publicly available comment data to build the commenter-overlap network. As a result, it excluded silent viewing patterns not captured through comments, which may overlook less interactive but influential content. Future research should integrate exposure metrics (eg, views and watch time) with survey or interview data to capture the full spectrum of engagement.

Another limitation is the lack of reliable demographic data on commenters, restricting assessment of engagement heterogeneity by age, gender, or location and limiting analysis of minority group vulnerability. We also could not confirm whether repeated commenter appearances reflected multiple accounts or bot activity, creating potential bias in interpreting edge density or centrality. This may produce an illusion of inflated interaction around certain channels or frame types, reducing the accuracy of network interpretations. Future research should combine network analysis with qualitative content analysis or targeted interviews to better capture user motivations, identities, and engagement authenticity.

### Conclusions

This study analyzed the multilayered discursive architecture of pro-ana content on YouTube by examining interactions among channels, frames, and networks. Content analysis showed that advocacy, criticism, and recovery frames varied systematically by channel ownership and subscriber scale. SNA revealed frame-based clustering and growing fragmentation over time. The ERGM estimated conditional probabilities of video-to-video connections based on shared commenters, offering insights into *who* spreads *which messages* and *how*.

A key finding is that meso-level individual channels, when delivering recovery or critical frames, foster cross-frame overlaps in participation that may disrupt pro-ana echo chambers. Theoretical contributions include: (1) conceptualizing pro-ana discourse as a “fragmented, refracted public” rather than a unified echo chamber, (2) advancing understanding of boundary construction and bridging mechanisms in digital public spheres, and (3) demonstrating the value of combining commenter-overlap network analysis with the ERGM for studying risk communication on digital platforms.

This study highlights the importance of *network-aware* health communication strategies that engage mid-scale individual channels as partners in disrupting harmful discourses and amplifying recovery frames. Public health institutions should collaborate with creators to increase message trust and reach while leveraging data-driven targeting to identify and intervene with at-risk groups. Commenter-based network analysis also provides a framework for understanding content flows and designing structural interventions that can open closed communities and promote exposure to health-supportive perspectives.

## Supplementary material

10.2196/77168 Multimedia Appendix 1 Video-level data.

10.2196/77168 Multimedia Appendix 2 Comment-level data.

## Abbreviations

ERGM: exponential random graph model
KPSS: Kwiatkowski-Phillips-Schmidt-Shin
OR: odds ratio
pro-ana: pro-anorexia
RQ: research question
SNA: social network analysis
